# Nfkbia-driven neuroinflammatory pathways mediate depression following spinal cord injury

**DOI:** 10.3389/fnmol.2025.1596649

**Published:** 2025-09-04

**Authors:** Aichun Yang, Guoyuan He, Yanling Song, Yujun Wen, Hechun Xia, Shenhong Gu

**Affiliations:** ^1^The First Clinical College, The First Affiliated Hospital, Hainan Medical University, Haikou, China; ^2^School of Clinical Medicine, Ningxia Medical University, Yinchuan, China; ^3^Ningxia Key Laboratory of Cerebrocranial Diseases, Ningxia Medical University, Yinchuan, China; ^4^Ningxia Key Laboratory of Stem Cell and Regenerative Medicine, Institute of Medical Sciences, General Hospital of Ningxia Medical University, Yinchuan, China; ^5^Department of Neurosurgery, General Hospital of Ningxia Medical University, Yinchuan, China

**Keywords:** spinal cord injury, depression, neuroinflammation, *Nfkbia*, bioinformatics

## Abstract

**Introduction:**

Spinal cord injury (SCI) frequently leads to severe motor impairments and psychological issues, particularly depression, which negatively affects overall quality of life. This study seeks to clarify the relationship between SCI and depression by employing a comprehensive approach that includes behavioral assessments, transcriptomic profiling, and molecular analyses.

**Methods:**

We established a weight-drop model of SCI and randomly assigned mice to Sham and SCI groups. Behavioral assessments included the Open Field Test (OP), Sucrose Preference Test (SP), and Tail Suspension Test (TS). We conducted transcriptomic analyses using datasets related to SCI and major depressive disorder (MDD) sourced from the GEO database. The hub gene, *Nfkbia*, was identified with Cytoscape software and validated through RT-PCR. Western blotting was utilized to measure the protein levels of IκB-*α* (encoded by *Nfkbia*) and phosphorylated p65 (p-p65). Additionally, we examined hippocampal histopathology and measured pro-inflammatory cytokines (IL-1β, IL-6, and TNF-α).

**Results:**

Following SCI, mice displayed abnormal behaviors in the OP, SP, and TS, suggesting the development of depression-like symptoms. In light of these observations, we analyzed publicly available transcriptomic datasets related to SCI and depression, identifying 16 common differentially expressed genes. Functional enrichment analysis showed that these genes were primarily associated with biological processes linked to inflammatory responses. We constructed a protein–protein interaction network that highlighted four potential key genes (*Nfkbia*, *Fkbp5*, *Sgk1*, and *Cdkn1a*). Subsequent molecular biology experiments confirmed that *Nfkbia* was downregulated after SCI, resulting in an increase in inflammatory factor production and the emergence of depression-like behaviors in mice.

**Discussion:**

Our results suggest that neuroinflammation plays a crucial role in the onset of depression after SCI. This is supported by the activation of the IκB/p65 signaling pathway and the dysregulation of inflammatory cytokines. These findings align with clinical observations of mood disorders in patients with SCI and reflect known patterns of inflammatory cytokine dysregulation. This study underscores the significance of anti-inflammatory treatments and comprehensive neuropsychiatric management strategies in the rehabilitation of SCI patients.

## 1 Introduction

Spinal cord injury (SCI) is a traumatic condition that affects the central nervous system (CNS) and is characterized by a high incidence rate, substantial healthcare costs, increased disability rates, and early onset (Hu et al., 2023). SCI can result from high-energy traumas such as falls, traffic accidents, and violent injuries, as well as from pathological conditions like infections, tumors, spinal degeneration, ischemia–reperfusion injury, and vascular anomalies (Alizadeh et al., 2019; Müller-Jensen et al., 2021; Hu et al., 2023). The pathophysiological processes of SCI involve both primary mechanical disruption and secondary injury cascades, leading to spinal cord compression, contusion, or transection, followed by hemorrhage, ischemia, and cellular necrosis. Secondary injury encompasses molecular, cellular, and biochemical responses triggered by the initial trauma, with neuroinflammation acting as a significant pathological factor (Li et al., 2020; Dolma and Kumar, 2021).

A key component of this neuroinflammatory cascade is the Nuclear factor-κB (NF-κB) signaling pathway, which is regulated by the inhibitory protein IκB-*α* (Ding and Chen, 2023). Under normal physiological conditions, Nfkbia mRNA is translated into IκB-α, which keeps the NF-κB complex in the cytoplasm. After neural injury, phosphorylation by IκB kinase (IKK) leads to the proteasomal degradation of IκB-α, allowing NF-κB to translocate to the nucleus and promote the transcription of pro-inflammatory mediators (Zhang et al., 2023). This molecular mechanism significantly contributes to the development of depression (Wang et al., 2014).

Rodent models of SCI have demonstrated activated microglia in the thalamus, hippocampus, and frontal cortex, indicating that post-SCI inflammation spreads beyond the spinal cord. Increased cerebral expression of pro-inflammatory cytokines related to microglial activation has been shown to influence behavioral outcomes (Wu et al., 2014a; Sun et al., 2016; Brakel and Hook, 2019). Clinical observations indicate a pathophysiological connection between major depressive disorder (MDD) and systemic inflammation, as seen by the increased risk of MDD in patients concurrently diagnosed with psoriasis (Lindqvist et al., 2009; Jensen et al., 2016). Additionally, the dynamic changes in neuroinflammation observed in depression animal models suggest that this process regulates hippocampal neurogenesis, further supporting the close relationship between hippocampal tissue and the onset of depression (Liu et al., 2022; Wu and Zhang, 2023). This association supports the hypothesis that individuals with SCI are at a greater risk for developing depression.

This study investigates the complex relationship between SCI and its comorbidity with depression. MDD is strongly associated with an increased risk of suicide, higher rates of urinary tract infections and pressure ulcers, longer hospital stays, decreased adherence to rehabilitation, lower levels of community engagement, and higher unemployment rates (Marwaha et al., 2023; Cui et al., 2024). While depressive symptoms following SCI may be similar to those found in the general population, post-SCI depression is characterized by increased clinical complexity and faster progression because of the altered inflammatory responses in the CNS after traumatic injury (Brakel and Hook, 2019). This divergence in pathophysiology highlights the need to clarify the key mechanisms that influence the onset and progression of depression in individuals with SCI.

However, our understanding of the connection between these two conditions is still limited. To address these knowledge gaps, this study employs a multifaceted approach that integrates behavioral assessments, transcriptomic profiling, and molecular analyses. We performed bioinformatics analyses on the GSE45006 and GSE183386 datasets from the Gene Expression Omnibus (GEO) to identify differentially expressed genes (DEGs) associated with SCI and MDD in murine models. Following this, we conducted Gene Ontology (GO) and KEGG functional enrichment analyses to clarify the biological significance of these DEGs.

We then constructed a protein–protein interaction (PPI) network and conducted topological analysis using Cytoscape to identify hub genes that play critical regulatory roles in the molecular pathways related to SCI-depression comorbidity. Concurrently, we performed behavioral assessments to evaluate depression-like behaviors in animal models. This integrated approach allows for a comprehensive examination of the behavioral and molecular changes that occur after SCI. The main goal of this study is to identify specific molecular pathways and hub genes involved in the comorbidity of SCI and MDD, thus providing a foundation for future therapeutic advancements.

## 2 Materials and methods

### 2.1 Animal preparation

Male C57BL/6 mice (28–32 g) at the age of 8–10 weeks were obtained from the Laboratory Animal Center of Ningxia Medical University. Male mice were selected for two primary reasons: first, the occurrence of SCI is notably more prevalent among men compared to women, making male mice a suitable model (Barbiellini Amidei et al., 2022). second, sex differences are known to influence neuroinflammatory responses (Khaksari et al., 2017). To eliminate sex-related variability, experiments were performed exclusively on male subjects (Zeng et al., 2023). All mice were housed under controlled conditions at a constant temperature (21 ± 3 °C) and humidity (50% ± 5%), with a 12-h light/dark cycle.

### 2.2 Experimental design

The mice were randomly divided into two groups: the Sham group and the SCI group. Behavioral experiments were conducted on days 31, 32, and 34, including the open field test (OP) (*n* = 3), the tail suspension test (TS) (*n* = 3), and the sucrose preference test (SP) (*n* = 3), to assess whether the mice exhibited depressive-like behaviors. Hippocampal tissue was then harvested for downstream analyses. Total RNA was extracted and analyzed by reverse transcription-quantitative PCR (RT-PCR); the sample size for validating the four candidate genes identified through bioinformatics analysis was *n* = 6, whereas all other RT-PCR assays were conducted with *n* = 3. Additional assays included western blotting (WB, *n* = 3), Nissl staining (*n* = 3), hematoxylin–eosin (HE) staining (*n* = 3), and immunofluorescence staining (*n* = 3).

### 2.3 SCI model *in vivo*

The SCI model was generated following established protocols as previously documented (Ni et al., 2019). The mice were subjected to anesthesia using isoflurane (2–4% for induction and 1.5% for maintenance) before performing a mid-back incision at the tenth thoracic level to expose the spinal cord. After performing a T10 laminectomy, a rod weighing 10 g was dropped from a height of 20 millimeters onto the spinal cord utilizing an impactor, with careful attention to prevent the application of excessive pressure. Subsequently, the suturing of the muscles and skin was carried out in a sequential manner, and manual bladder evacuation was conducted twice daily for 1 week until the restoration of normal bladder functionality was achieved. The body temperature of the animals were kept at a controlled temperature of 35 ± 1 °C utilizing a miniature heating pad. In the Sham group, a laminectomy procedure was conducted without inducing an SCI model.

### 2.4 WB

Hippocampal tissue samples were obtained, and protein extraction was performed utilizing a Total Protein Extraction Kit (KeyGEN Bio TECH, Nanjing, Jiangsu, China) in alignment with the guidelines stipulated by the manufacturer. Subsequently, the protein concentrations were determined using a BCA protein assay kit (KeyGEN Bio TECH, Nanjing, Jiangsu, China). Identical volumes of protein samples were subjected to separation by SDS-PAGE using gels with concentrations ranging from 10 to 12%. The proteins underwent a transfer process onto polyvinylidene difluoride (PVDF) membranes. Blocking was conducted utilizing a freshly prepared solution of 5% skim milk for a duration of 1 to 2 h, succeeded by an overnight incubation at 4 °C with the primary antibodies: IκB-*α* (dilution 1:2000; Abcam, Cat. No. ab32518, Cambridege, Massachusetts, United States), p-IκB-α (dilution 1:1000; Abcam, Cat. No. ab92700, Cambridege, Massachusetts, United States), p-p65(dilution 1:1000; Invitrogen, Cat. No. MA5-15160, Carlsbad, California, United states), and beta actin (dilution 1:10000; Affinity, Cat. No. AF7018, Nanjing, Jiangsu, China). Finally, horseradish peroxidase (HRP)-conjugated secondary antibodies were introduced to the membranes and incubate at room temperature for 1 h. The visibility of the blotted protein bands was achieved through the application of an enhanced chemiluminescence (ECL) kit (KeyGEN Bio TECH, Nanjing, Jiangsu, China), and the quantitative assessment was performed utilizing ImageJ software (National Institutes of Health, Bethesda, MD, USA), with normalization against *β*-actin levels.

### 2.5 RT-PCR

To validate the gene expression alterations identified through transcriptomic analysis, a RT-PCR assay was conducted. Total RNA was isolated from the hippocampus using TRIzol reagent.

The quality of the RNA was evaluated utilizing a Thermos Nanodrop Analyzer, with an A260/A280 ratio between 1.8 and 2.0 indicating good RNA purity. Reverse transcription was conducted utilizing a Light Cycle 96 Real-time PCR system. The relative expression levels of relative genes were determined employing the 2^-ΔΔCt^ methodology, utilizing β-actin as the internal control. Gene expression analyses were performed at three time points. The sequences of the primers can be found in Table 1.

**Table 1:** Primers utilized in this study.

Gene	Primers	Sequence
*Nfkbia*	Forward primers	TGAAGGACGAGGAGTACGAGC
	Reverse primers	TTCGTGGATGATTGCCAAGTG
*Cdkn1a*	Forward primers	CCTGGTGATGTCCGACCTG
	Reverse primers	CCATGAGCGCATCGCAATC
*Sgk1*	Forward primers	CTGCTCGAAGCACCCTTACC
	Reverse primers	TCCTGAGGATGGGACATTTTCA
*Fkbp5*	Forward primers	TGAGGGCACCAGTAACAATGG
	Reverse primers	CAACATCCCTTTGTAGTGGACAT
*IL-1β*	Forward primers	GCAACTGTTCCTGAACTCAACT
	Reverse primers	ATCTTTTGGGGTCCGTCAACT
*IL-6*	Forward primers	TAGTCCTTCCTACCCCAATTTCC
	Reverse primers	TTGGTCCTTAGCCACTCCTTC
*TNF-α*	Forward primers	CCCTCACACTCAGATCATCTTCT
	Reverse primers	GCTACGACGTGGGCTACAG

### 2.6 Immunofluorescence staining

The immunofluorescence staining protocol described by Zhang et al. (2025) was performed to achieve precise image visualization. At first, hippocampal brain sections were subjected to overnight incubation at 4 °C with a rabbit anti-IκB-*α* antibody (dilution 1:50; Abcam, Cambridge, Massachusetts, United States). Following a wash with phosphate-buffered saline, a mouse anti-Iba-1 antibody (dilution 1:100; Abcam, Cambridge, Massachusetts, United States) was utilized and incubated overnight. Subsequently, after an additional PBS wash, brain sections were permitted to incubate at ambient temperature for 1 h with goat anti-rabbit IgG (dilution 1:50; Proteintech, Wuhan, Hubei, China) and goat anti-mouse IgG (dilution 1:200; Proteintech, Wuhan, Hubei, China). Thereafter, the sections were allowed to incubate at room temperature for a duration of 5 min DAPI (Biosharp, Hefei, Anhui, China). Eventually, the sections that had been stained were inspected using a BX43F fluorescence microscope (Olympus, Japan), and images were obtained at 400 × magnification.

### 2.7 Nissl staining

To evaluate neuronal cell death, Nissl staining was conducted following established protocols from previous studies. Brain specimens underwent a process of fixation, dehydration, embedding in paraffin, and were subsequently sectioned to a thickness of 10 μm. The tissue samples were subjected to a dewaxing process using xylene, repeating the procedure three times for a duration of 5 min each time. Subsequently, the specimens were treated sequentially with anhydrous ethanol for a duration of 5 min, 90% ethanol for 2 min, 70% ethanol for another 2 min, and distilled water for 2 min. The specimens underwent Nissl staining for a duration of 10 min, followed by two brief rinses with distilled water. Next, the specimens underwent a dehydration process using 95% ethanol, applied twice for a duration of 2 min per treatment. Subsequently, a clearing procedure was performed with xylene, repeated twice for 5 min each time. Finally, the specimens were mounted utilizing neutral gum. Under light microscopy, normal neurons displayed large cell bodies with abundant cytoplasm. Conversely, damaged neurons exhibited reduced cell body size and the presence of numerous vacuoles.

### 2.8 HE staining

HE staining was conducted using an HE staining kit (G1120, Solarbio, Beijing, China). In summary, brain sections were stained with Mayer’s hematoxylin and eosin. After a 50-s exposure to eosin and subsequent dehydration in 95 and 100% ethanol, the sections were cleared with xylene and mounted. Images were captured by microscope (Olympus, Tokyo, Japan).

### 2.9 Op

The OP is widely used in neurological research for its simplicity and effectiveness in assessing depression-like behaviors in murine models. In this experiment, mice were positioned at the center of a box with dimensions of 50 × 50 × 35 cm and were permitted to explore the environment for a duration of 5 min. The lighting condition in the experimental area was set at 30 lux. The behavioral patterns of each mouse were captured utilizing a video tracking system provided by RWD Life Science Co., Ltd.

### 2.10 TS

For the TS, mice were hung by bands attached to the edges of a 20-cm-high platform. The duration of immobility during a 5-min assessment period was recorded utilizing a video recording device (RWD Life Science Co., Ltd.). Immobility was defined as the lack of movement, with the exception of whisker movements and respiratory activity.

### 2.11 SP

The SP was conducted under controlled conditions. Prior to the main experiment, mice were given access to two containers, each filled with a 1% sucrose solution (Sinopharm, Shanghai, China) for a duration of 24 h. The two bottles were placed on opposite sides of the cage, ensuring that the distance from the nest area to each bottle was identical. Subsequently, one of the containers was substituted with pure water, allowing the mice to acclimatize for an additional 24-h period. Following this acclimation phase, the locations of the two containers were exchanged. Prior to the assessment, the mice underwent a 24-h deprivation of both food and water. After this deprivation, they were offered pre-determined quantities of the 1% sucrose solution and pure water during a 3-h testing interval. The residual volumes in each container were measured post-assessment to determine the sucrose preference percentage, which was calculated using the formula: sucrose preference (%) = sucrose consumption / (sucrose consumption + water consumption) × 100%.

### 2.12 Data sources

Gene expression datasets GSE45006 and GSE183386 were downloaded from the GEO database using the search terms “Spinal cord injury” and “Depression.” The corresponding annotation platforms for these datasets are GPL1355 and GPL6247, respectively.

### 2.13 Screening of DEGs

The GEO2R online platform was utilized to analyze raw data for the purpose of identifying DEGs between SCI and MDD. DEGs were filtered based on the criteria of |log_2_*FC*| > 0.5 and *p* < 0.05, yielding a comprehensive list of genes (Supplementary Table S1). Volcano plots for DEGs in datasets GSE45006 and GSE183386 were accomplished utilizing the “Ggplot2” package within the R, whereas heatmaps were produced with the “ComplexHeatmap” package (Gu et al., 2016). The intersection of DEGs from GSE45006 and GSE183386 was identified, and Venn diagrams were constructed using the “Ggplot2”and “VennDiagram” packages.

### 2.14 Enrichment analysis of common DEGs

To investigate the notably enriched biological functions and signaling pathways associated with these DEGs, the “ClusterProfiler” package (Wu et al., 2021) was used to perform GO and KEGG analyses on the common DEGs from GSE45006 and GSE183386. Bubble plots summarizing the enrichment analysis results were generated using the “Ggplot2” package.

### 2.15 Analysis and screening the hub genes

The DEGs identified in the study were subsequently submitted to the STRING database to facilitate the construction of a PPI network through the use of the available integrated analysis tools. Subsequently, the results were imported into Cytoscape software, where the MMC algorithm in the cytoHubba plugin was used to identify the top four hub genes based on interaction scores.

### 2.16 Statistical analysis

The data are presented as the mean ± standard deviation (SD). Statistical differences were assessed using Student’s t-test for all comparisons in this study. All statistical analyses were conducted using GraphPad Prism (version 9.3). A *p*-value < 0.05 was considered statistically significant.

## 3 Results

### 3.1 SCI induces depressive-like behaviors in C57BL/6 mice

**Figure 1:**
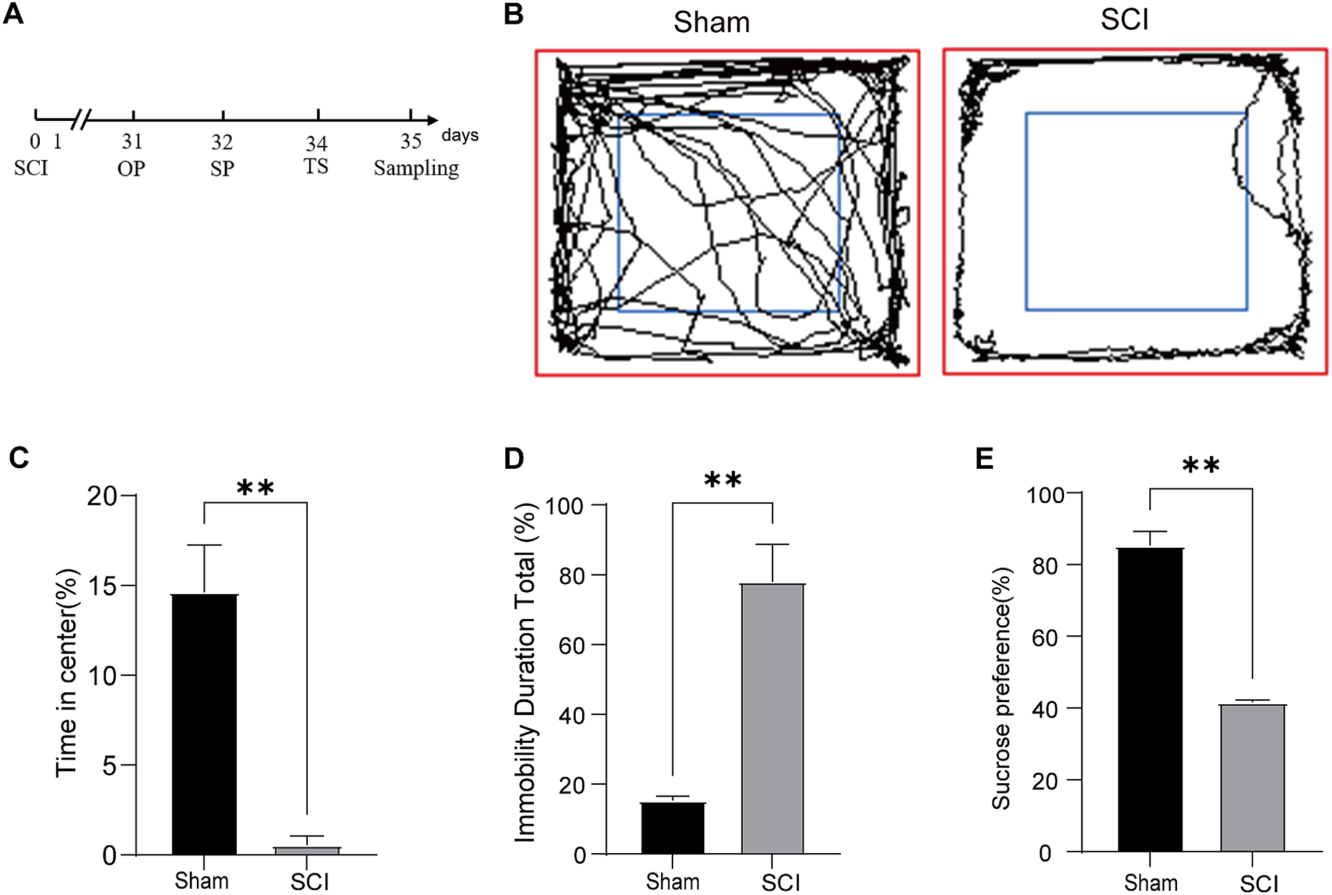
C57BL/6 mice exhibit depression-like behaviors following SCI. **(A)** A schematic representation of the experimental design is provided. **(B)** Representative traces from the OP test; **(C)** OP test analysis: percentage of time spent in the center by Sham and SCI groups; **(D)** TS analysis: percentage of immobility time in Sham and SCI groups. **(E)** SP test analysis: percentage of sucrose preference (Mean ± SD, *n* = 3, **p < 0.01).

To evaluate the presence of depressive symptoms, we conducted standardized behavioral evaluations, including the OP, SP, and TS. As illustrated in Figure 1A, the SCI model was established on day 0, followed by OP, SP, and TS on days 31, 32, and 34, respectively. The OP results revealed significant behavioral changes, with SCI mice exhibiting reduced exploration of the center zone compared to Sham controls (Figures 1B,C). Although the total distance traveled did not significantly differ between the SCI and Sham groups during the OP, the SCI group showed a reduction in distance traveled (Supplementary Figure S1A). Furthermore, the average velocity of the SCI group was significantly lower in the OP (Supplementary Figure S1B). These findings suggest that the reduced exploration in the center zone during the OP may more accurately reflect the presence of depressive-like behaviors in the SCI group. The SP results indicated significant anhedonia in the SCI mice (Figure 1E). Similarly, in the TS, the SCI mice exhibited prolonged immobility following the injury (Figure 1D). Due to motor impairments in mice with SCI, we are unable to determine whether the increased immobility observed in the TS is caused by depressive-like behavior or impairments in motor function. Therefore, we combined the results from the OP and the SP to comprehensively assess whether the mice exhibited depressive-like behaviors. Ultimately, we concluded that mice exhibit significant depressive-like phenotypes following SCI.

### 3.2 Transcriptomic profiling reveals conserved molecular signatures

**Figure 2:**
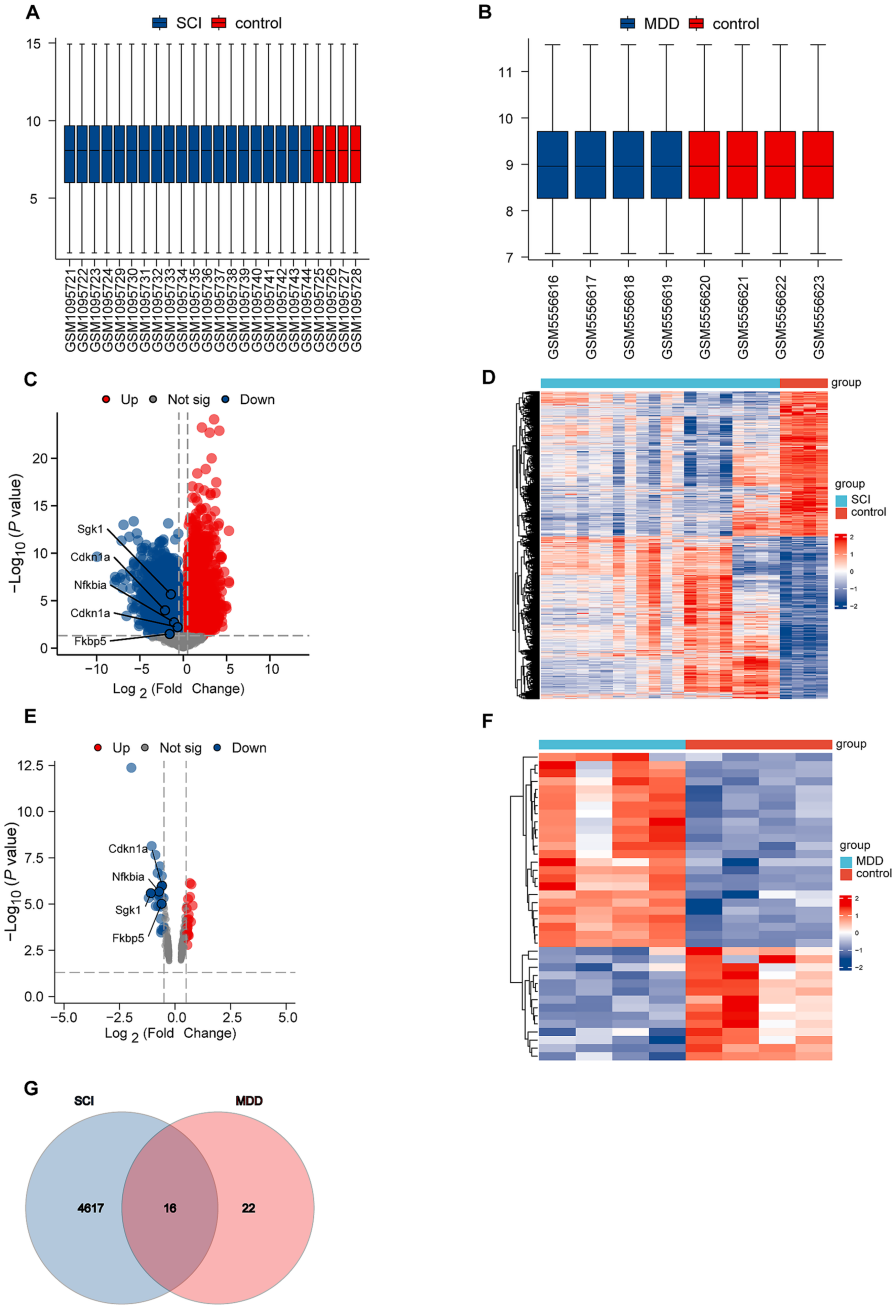
Identification of DEGs. **(A,B)** Box plots. **(C)** Volcano plot showing DEGs in dataset GSE45006. **(D)** Heatmap illustrating DEGs in dataset GSE45006. **(E)** Volcano plot showing DEGs in dataset GSE183386. **(F)** Heatmap illustrating DEGs in dataset GSE183386. **(G)** Venn diagram showing 16 overlapping DEGs between datasets GSE45006 and GSE183386.

To investigate the molecular factors associated with the comorbidity of SCI and depression, we compared the transcriptomes from SCI (GSE45006) with those from depression (GSE183386). Following normalization, as illustrated in Figures 2A,B for the GSE45006 and GSE183386 datasets, respectively, the distribution of the two sample sets met standard criteria, confirming that the microarray data were of high quality. Utilizing the “ggplot2” package, we identified 4,633 DEGs in the GSE45006 dataset and 38 DEGs in the GSE183386 dataset, with volcano plots illustrating these findings in Figures 2C,E, respectively. The “ComplexHeatmap” package was employed to generate heatmap visualizations of these DEGs, as shown in Figures 2D,F.

Intersection analysis of DEGs from both datasets using the ggplot2 and “VennDiagram” packages identified 16 co-expressed DEGs, illustrated in a Venn diagram (Figure 2G). These shared DEGs include *Nfkbia* (NF-κB inhibitor alpha); *Fkbp5* (FK506-binding protein 5); *Acer2* (alkaline ceramidase 2); *Cd180* (CD180 molecule); *Cdkn1a* (cyclin-dependent kinase inhibitor 1a); *Errfi1* (ERBB receptor feedback inhibitor 1); D*dit4* (DNA damage-inducible transcript 4); *Gpr149* (G protein-coupled receptor 149); *Mmp14* (matrix metallopeptidase 14); *Pcdh20* (protocadherin 20); *Pla2g3* (phospholipase A2 group III); *Psmb9* (proteasome 20S subunit beta 9); *Sgk1* (serum/glucocorticoid-regulated kinase 1); *Pvalb* (parvalbumin); *Slc18a3* (solute carrier family 18 member a3); and *Slc5a7* (solute carrier family 5 member 7).

### 3.3 Functional annotation reveals neuroinflammatory pathways

**Figure 3:**
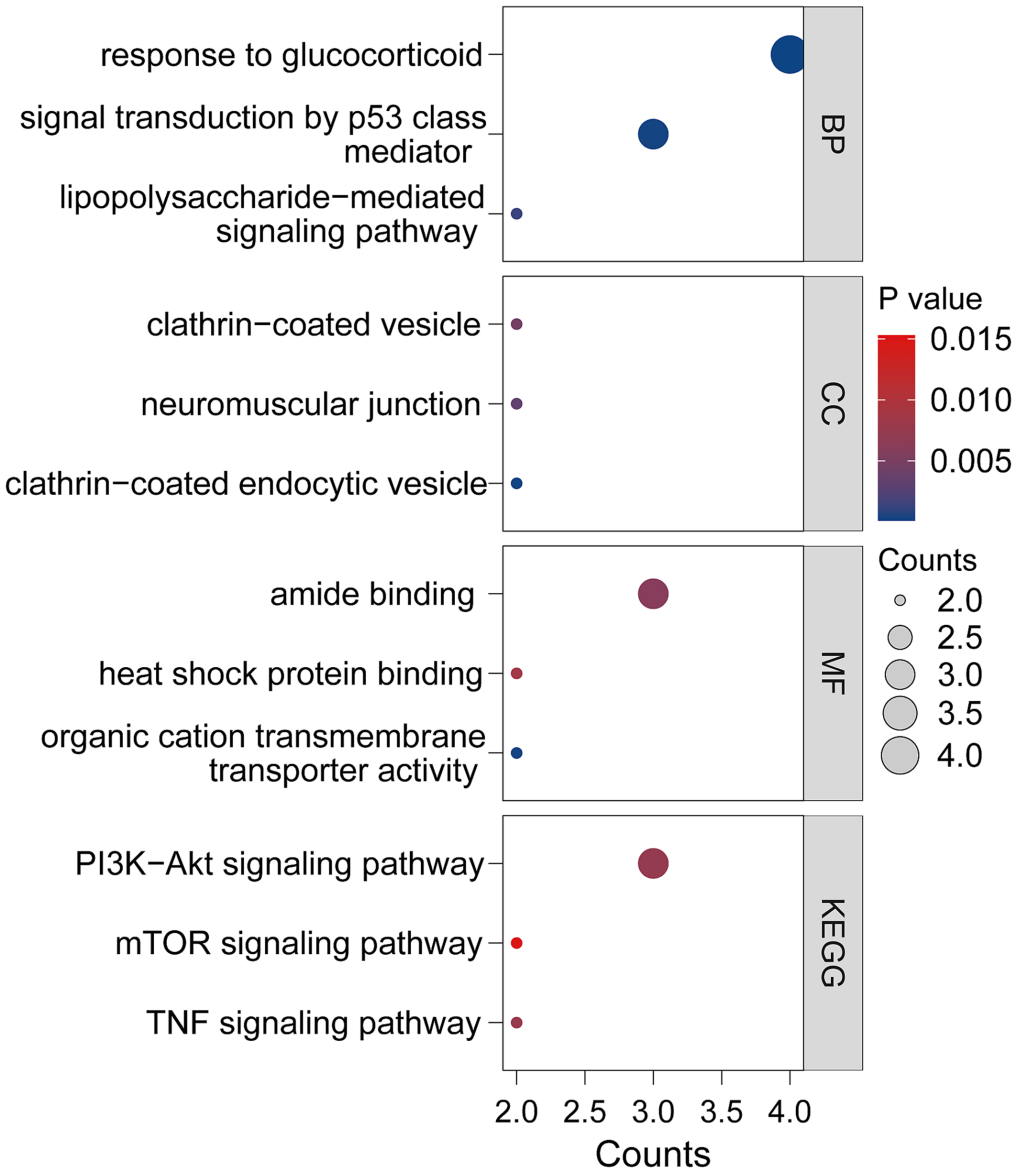
GO and KEGG pathway enrichment analysis results for 16 common genes.

Both GO and KEGG analyses confirmed a significant association between the 16 co-expressed DEGs and various signaling pathways (Figure 3). These DEGs were classified into three main functional categories: biological processes (BP), molecular functions (MF), and cellular components (CC). The key BP comprised “Response to glucocorticoid,” “Cellular response to glucocorticoid stimulus” and “Response to corticosteroid.” MF analysis identified “Heat shock protein binding” and “Ubiquitin protein ligase binding.” The CC category mainly included “Clathrin-coated endocytic vesicle,” “Neuromuscular junction,” and “Clathrin-coated vesicle.” KEGG pathway analysis pinpointed key inflammatory cascades, such as “PI3K-Akt signaling pathway.” Collectively, the data indicated that these DEGs predominantly participate in neuroinflammatory regulation.

### 3.4 Protein interaction network identifies hub genes

**Figure 4:**
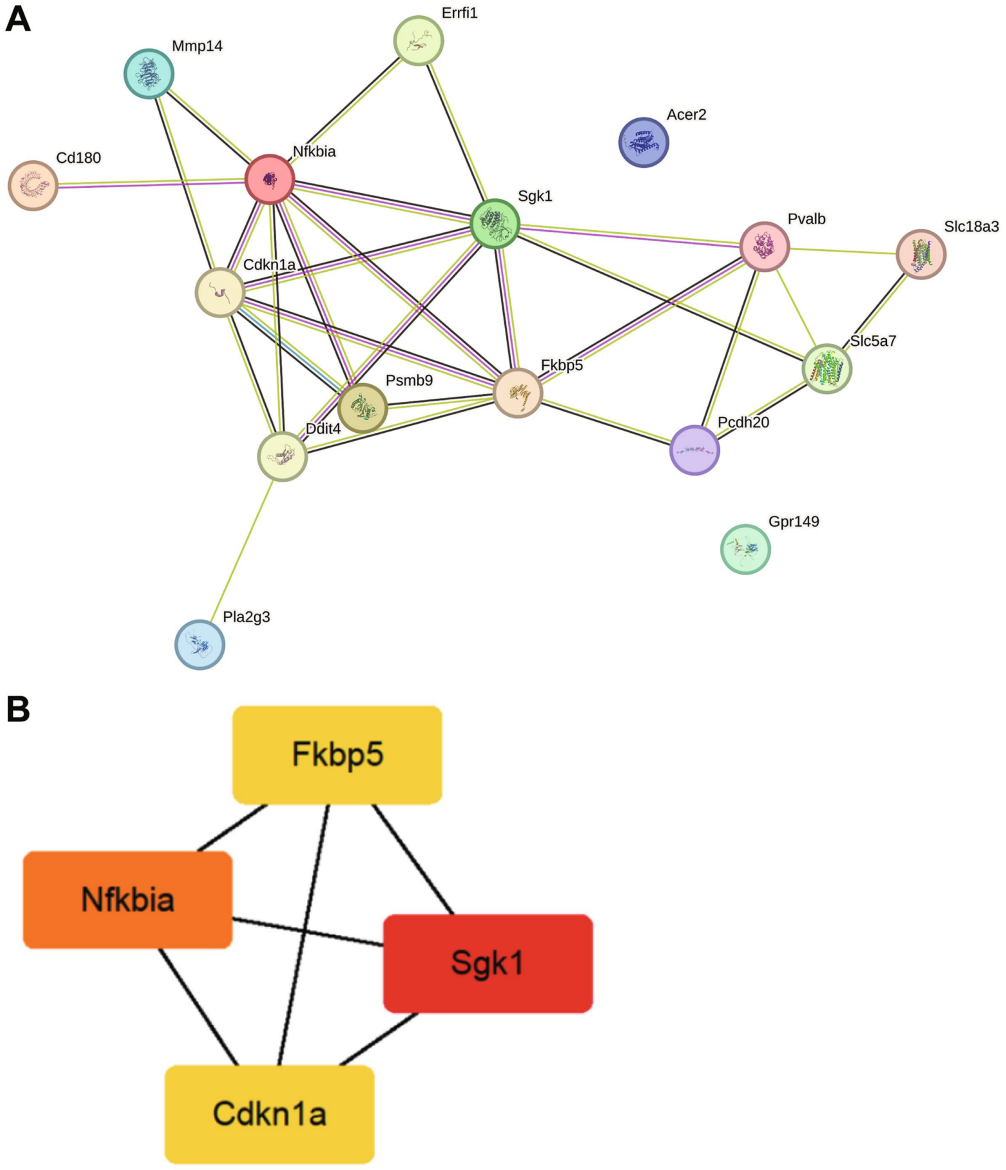
Interactions among the DEGs. **(A)** PPI network networks based on the STRING database, showing interactions among shared genes between SCI and MDD. **(B)** Top four hub genes identified using Cytoscape.

To explore protein interactions, we constructed a PPI network consisting of 36 common genes, utilizing the STRING database. The PPI network analysis (confidence coefficient = 0.15) revealed 16 hub targets, with 28 edges, an average node degree of 3.5, and an average clustering coefficient of 0.633 (Figure 4A). We imported the PPI network into Cytoscape, visualized it using the Cytohubba plugin, and analyzed it with the MCC algorithm to evaluate its overall topological structure. Based on node significance, the four key hub genes identified were *Nfkbia*, *Fkbp5*, *Sgk1*, and *Cdkn1a* (Figure 4B). These candidate genes are annotated in the volcano plots (Figures 2C,E).

### 3.5 Experimental validation of hub genes

**Figure 5:**
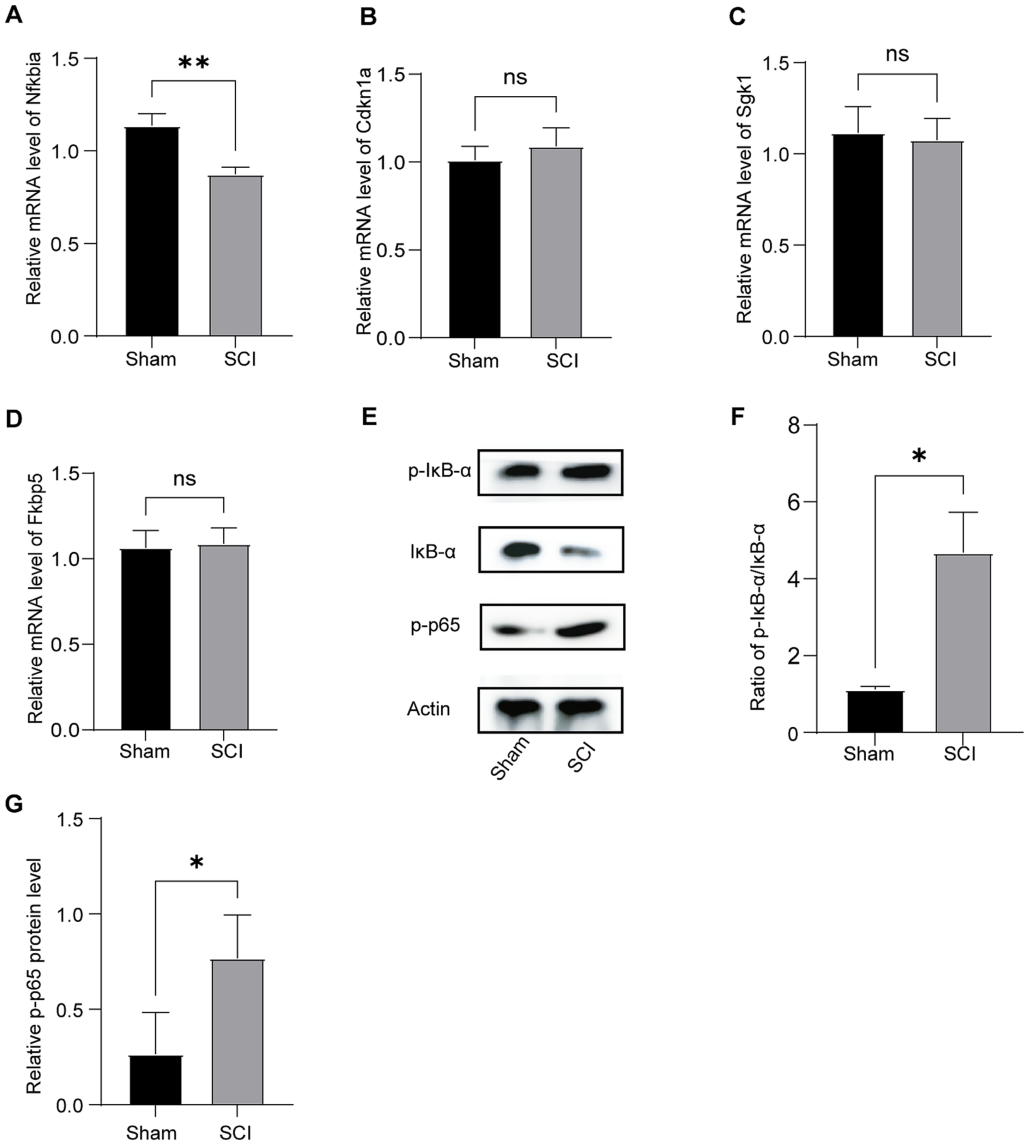
Validation of mRNA and protein expression levels of hub genes in hippocampal tissue of mice following SCI using RT-PCR and WB. **(A–D)** The expression levels of mRNA for the four key hub genes were quantified (*n* = 6). **(E)** Representative WB bands showing p-IκB-α, IκB-α, and p-p65 expression in different groups (*n* = 3). **(F)** The ratio of p-IκB-α to IκB-α was analyzed. **(G)** A semi-quantitative analysis of the expression levels of p-p65 across different groups was conducted (Mean ± SD, * *p* < 0.05 and ** *p* < 0.01).

To confirm the transcriptomic findings, we performed RT-PCR and WB analyses on hippocampal tissues obtained from Sham and SCI mice. As shown in Figure 5A, SCI mice exhibited significant downregulation of Nfkbia mRNA compared to Sham controls, while *Fkbp5*, *Sgk1*, and *Cdkn1a* did not show significant changes (Figures 5B–D).

In terms of mechanism, canonical NF-κB activation involves phosphorylated IκB-*α*, which releases the NF-κB (p50/p65) complex, allowing it to translocate to the nucleus and initiate the transcription of pro-inflammatory cytokines such as IL-1β, IL-6, TNF-α. This process is counter-regulated by the resynthesis of IκB-α, encoded by *Nfkbia* (Taniguchi and Karin, 2018). Next, we evaluated the expression of IκB-α and phosphorylated IκB-α (p-IκB-α) using WB analysis. The WB results showed an increased p-IκB-α/IκB-α ratio in the SCI group compared to the Sham group (Figures 5E,F), indicating prolonged activation of NF-κB signaling. We then assessed the expression of p-p65 using WB analysis. Semi-quantitative analysis of p-p65, the active form of the NF-κB transcription factor, revealed a significant increase in SCI mice compared to Sham controls (Figure 5G). Taken together, these findings identify *Nfkbia* as a key regulator of the comorbidity between SCI and depression.

### 3.6 Neuroinflammatory cascade and histopathological changes

**Figure 6:**
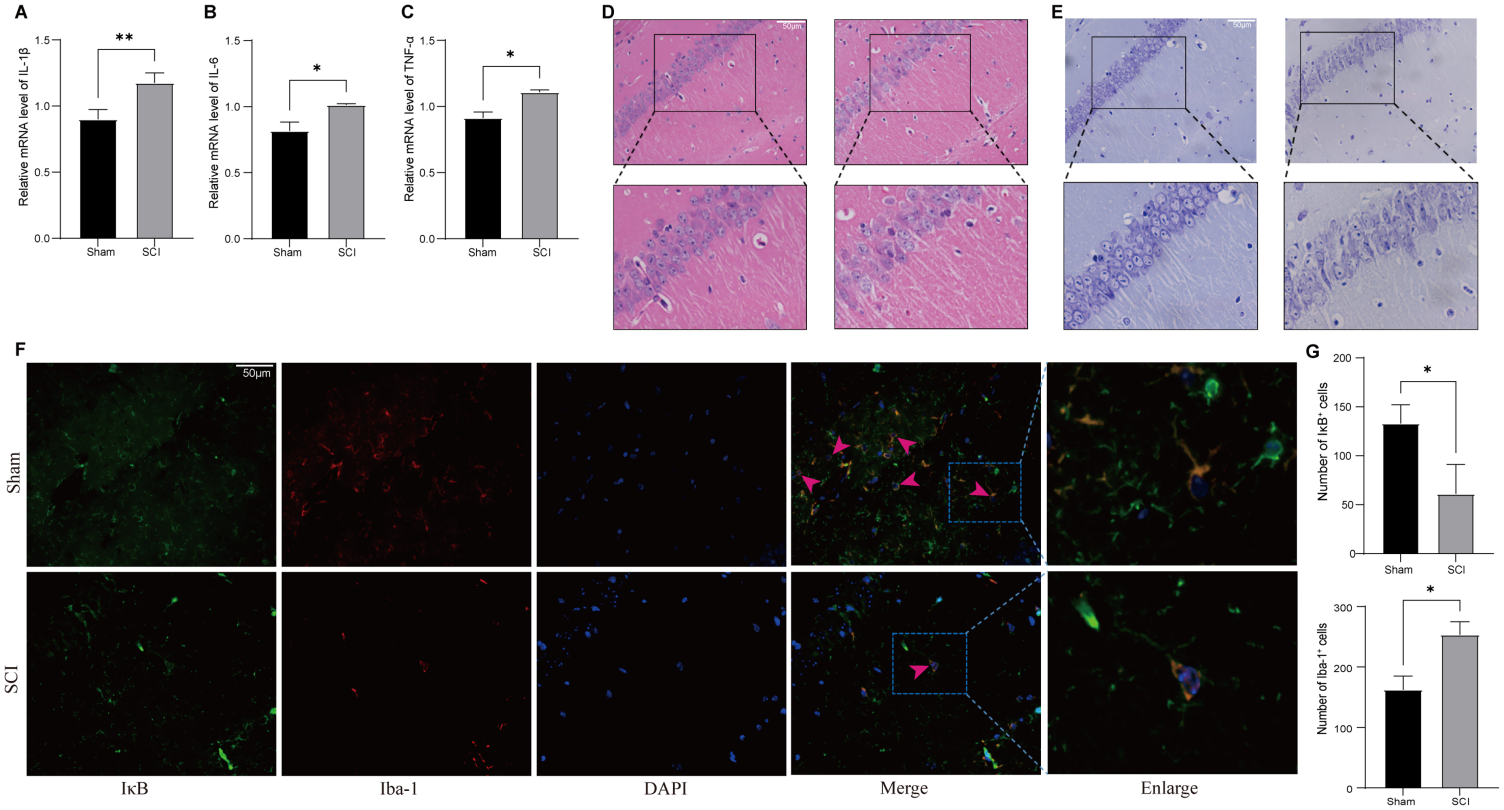
Neuroinflammatory alterations and neuronal damage in hippocampal tissue following SCI. **(A–C)** Significant alterations in inflammatory mediators were observed in hippocampal tissue following SCI using RT-PCR. **(D,E)** Histological analysis of the hippocampus using HE and Nissl staining. **(F,G)** Representative immunofluorescence images of IκB-α and Iba-1 in hippocampal regions across Sham and SCI group. Red arrowheads indicate regions of IκB-α and microglia co-expression. Boxed areas are shown magnified. Quantification of IκB-α- and Iba-1-positive cells per field, as determined with ImageJ, is presented in the right panel (Mean±SD, *n* = 3, * *p* < 0.05 and ** *p* < 0.01), scale bars = 50 μm.

Supporting our molecular findings, RT-PCR analysis revealed increased levels of pro-inflammatory cytokines in SCI mice, including IL-1β (Figure 6A), IL-6 (Figure 6B), and TNF-*α* (Figure 6C), compared to Sham controls. The observed cytokine expression patterns correlate with the NF-κB activation profile shown in Figure 5G, highlighting the functional relationship between IκB-α phosphorylation and the production of inflammatory mediators.

Histopathological examination using HE staining revealed notable changes in the hippocampal CA1 region of mice with SCI-depression comorbidity, including partial neuronal loss, cytoplasmic vacuolation, and disrupted alignment of pyramidal cells (Figure 6D). Nissl staining demonstrated that Sham mice retained normal cytoarchitecture, exhibiting densely packed CA1 pyramidal neurons with intact Nissl bodies (Figure 6E). In contrast, SCI mice exhibited significant neurodegeneration characterized by disrupted neuronal arrangement, enlarged intercellular gaps, shrunken neuronal cell bodies, and a marked decrease in cytoplasmic Nissl body density. Immunofluorescence microscopy indicated that IκB-α co-localized with the microglia marker Iba-1 (Figure 6F, red arrowheads). Additionally, IκB-α levels were reduced in the SCI group compared to the Sham group, as highlighted by the red arrowheads in Figure 6F. This spatial distribution supports the increased p-IκB-α/IκB-α ratio observed in Figure 5F. Figure 6G illustrates the number of IκB-α and Iba-1 positive cells in both the SCI and Sham groups.

## 4 Discussion

This study provides new insights into the neuroinflammatory mechanisms underlying depression related to SCI, integrating behavioral, transcriptomic, and molecular evidence. Two key findings are highlighted: (1) *Nfkbia* acts as a crucial hub gene connecting the pathophysiology of SCI and MDD, and (2) neuroinflammation likely plays a significant role in depression associated with SCI.

### 4.1 Neuroinflammation and depression in SCI

The prevalence of depression among individuals with SCI is about twice as high as that in the general population (Brakel and Hook, 2019). This trend is also evident in rodent models, where subjects with SCI demonstrate increased depressive-like behaviors, assessed through various experimental measures in this study, including the OP, SP, and TS. Although psychosocial and socioeconomic factors have traditionally been viewed as the main causes of depression after SCI, recent preclinical evidence indicates that biological mechanisms also significantly contribute to its development. In a study by Brakel et al., individuals with higher levels of pro-inflammatory cytokines both before and after SCI were more likely to develop depressive symptoms (Brakel et al., 2021). Similarly, the study found that ongoing peripheral and central inflammation significantly affects psychological health following SCI (Maldonado-Bouchard et al., 2016). Inflammation may serve as either a cause or a biomarker for the onset of depression-like behaviors after SCI.

Consistent with previous studies, we observed significant differences in pro-inflammatory cytokine expression in the SCI group (Wu et al., 2014b; Brakel and Hook, 2019; Santo et al., 2019). The levels of pro-inflammatory cytokines in the hippocampal tissue of the SCI group were elevated compared to those in the sham group. This suggests that inflammation extends beyond the primary injury site, as the brain also produces pro-inflammatory cytokines. Following SCI, the brain also expresses pro-inflammatory cytokines linked to activated microglia, which release cytotoxic and pro-inflammatory factors. This leads to neurotoxicity in the surrounding environment and triggers behavioral changes related to cell death (Wu et al., 2014a). Despite these findings, there are currently no specific treatment options available for depression following SCI. A thorough exploration of the molecular pathways involved in SCI-specific depression is essential for understanding the condition and identifying potential treatment options.

### 4.2 *Nfkbia’s* role in neuroinflammation and SCI-related depression

We identified potential co-expressed genes and pathways that interact with SCI and depression using bioinformatics analysis. We subsequently screened four hub genes from the protein–protein interaction (PPI) network: *Nfkbia*, *Fkbp5*, *Sgk1*, and *Cdkn1a*. RT-PCR analysis of these four genes showed that only Nfkbia mRNA was significantly downregulated in the SCI group compared to the sham group; this finding was further validated by WB analysis. In sepsis-associated encephalopathy, ALKBH5 attenuates inflammation by slowing the degradation of NFKBIA mRNA, thereby increasing NFKBIA protein levels (Ye et al., 2025). KDM5B enhances macrophage-mediated inflammatory responses by repressing *Nfkbia* transcription (Zhang et al., 2023). This suggests that *Nfkbia* is important for regulating inflammatory responses. Importantly, the *Nfkbia* gene encodes IκB-*α*, which maintains NF-κB in an inactive cytoplasmic state under resting conditions (Yu et al., 2020). Among the NF-κB dimer family, the p50/p65 heterodimer, the most common isoform in mammalian cells, serves as the primary driver of inflammatory signaling (Taniguchi and Karin, 2018). IκB molecules are phosphorylated in response to inflammatory cytokines, bacterial or viral products, and various forms of cellular stress (Israel, 2010). This process releases the p50/p65 heterodimer, enabling its translocation into the nucleus to activate the transcription of inflammatory genes. When NF-κB is activated, it may promote the expression of the IκB-*α* gene through a direct mechanism. This process ensures that cells can rapidly synthesize IκB-α protein, effectively regulating the activity of NF-κB (Sun et al., 1993). Additionally, studies have shown that the circadian rhythm gene NFIL3 can activate the NF-κB signaling pathway by inhibiting the transcription of *Nfkbia* (Yang et al., 2022). This finding further emphasizes the complexity of *Nfkbia* regulation and suggests that further research is needed to gain a deeper understanding of this mechanism. In our study, SCI mice exhibited: (1) an increased p-IκB-α/IκB-α ratio and elevated p-p65 levels, and (2) a reduction in the intensity of IκB-α immunofluorescence. These findings provide compelling evidence that the reduction of *Nfkbia* following SCI activates the IκB/NF-κB axis, thereby promoting neuroinflammation and ultimately mediating depression-like behaviors.

Investigations of neuroinflammatory pathways have yielded significant insights into the mechanisms driving the comorbidity of SCI and depression. Our experimental validation of NF-κB pathway activation (evidenced by an increased p-IκB-α/IκB-α ratio and enhanced p-p65 expression) provides direct molecular evidence that SCI-induced neuroinflammation underlies the comorbidity of depression. Although the KEGG analysis did not specifically indicate the involvement of the NF-κB pathway, the observed enrichment of the PI3K/Akt signaling pathway suggests a bidirectional regulatory interaction with NF-κB signaling. This finding is consistent with prior research showing mutual interactions between the PI3K/Akt and NF-κB pathway (Taniguchi and Karin, 2018). Specifically, the PI3K/Akt pathway promotes NF-κB nuclear translocation through IKK-dependent IκB phosphorylation. This pathway crosstalk creates an inflammatory amplification loop, consistent with the observed increases in cytokines (IL-1β, IL-6, TNF-α). Identifying these pathways lays a foundation for future research. This research aims to develop targeted interventions that address both the neuroinflammatory and psychological aspects of SCI-induced depression.

Histopathological examinations confirm the behavioral and molecular findings, revealing significant neuronal loss and alterations in the CA1 region of the hippocampus in SCI mice. The observed neurodegenerative changes were associated with behavioral outcomes, establishing a clear link between physical trauma and the neurobiological basis of depression. This finding is particularly relevant, as the hippocampus is crucial for mood regulation, and its dysfunction may exacerbate depressive symptoms in patients with SCI. These observations highlight the necessity for histopathological evaluations in future research to clarify the neurobiological mechanisms underlying SCI and depression, thereby guiding neuroprotective strategies that protect neuronal integrity and function in vulnerable populations.

### 4.3 Limitations of the study and future directions

This study identified key genes associated with depressive-like behaviors following SCI through bioinformatics and validated the potential of *Nfkbia* as a diagnostic and therapeutic biomarker for these behaviors through *in vivo* experiments, thereby providing a theoretical foundation for clinical diagnosis and treatment. However, there are some limitations to this study. First, our behavioral tests were conducted at only one late time point, which limits the breadth of the findings. Second, although we explored the role of *Nfkbia* in neuroinflammation and depression related to SCI, there was a lack of in-depth functional analysis. Additionally, we only investigated the IκB/NF-κB signaling pathway in microglia, leaving its presence in neurons or astrocytes unexplored. Finally, the sample size in this study was relatively small. Future studies will adopt a longitudinal design to characterize the temporal dynamics of SCI-associated depressive symptoms, deepen our functional understanding of *Nfkbia*, and systematically compare the contribution of the IκB/NF-κB pathway in microglia, astrocytes, and neurons to the behavioral phenotype. As our understanding of these interrelationships continues to deepen, targeted therapeutic strategies addressing both the physiological and psychological aspects of SCI may significantly improve patient outcomes and quality of life.

## 5 Conclusion

This research offers valuable insights into the molecular and behavioral impacts of depression resulting from SCI. Our findings reveal a conserved molecular signature linking SCI and depression, highlighting crucial neuroinflammatory pathways, especially the IκB/NF-κB signaling pathway, and identifying *Nfkbia* as a promising therapeutic target. The integration of behavioral assessments, transcriptomic profiling, and molecular analyses emphasizes the intricate relationship between SCI and depression, providing guidance for future research on targeted therapeutic interventions. In conclusion, *Nfkbia* and its associated inflammatory signaling pathways play significant roles in depression. Additionally, *Nfkbia* has the potential for early prediction of depression induced by SCI.

## Data Availability

The datasets presented in this study can be found in online repositories. The names of the repository/repositories and accession number(s) can be found below: https://www.ncbi.nlm.nih.gov/, GSE45006 and GSE183386.

## References

[ref1] Alizadeh A. Dyck S. M. Karimi-Abdolrezaee S. (2019). Traumatic spinal cord injury: an overview of pathophysiology, models and acute injury mechanisms. Front. Neurol. 10:282. doi: 10.3389/fneur.2019.00282, PMID: 30967837 PMC6439316

[ref2] Barbiellini Amidei C. Salmaso L. Bellio S. Saia M. (2022). Epidemiology of traumatic spinal cord injury: a large population-based study. Spinal Cord 60, 812–819. doi: 10.1038/s41393-022-00795-w, PMID: 35396455 PMC8990493

[ref3] Brakel K. Aceves M. Garza A. Yoo C. Escobedo G. Jr. Panchani N.. (2021). Inflammation increases the development of depression behaviors in male rats after spinal cord injury. Brain Behav Immun Health 14:100258. doi: 10.1016/j.bbih.2021.100258, PMID: 34589764 PMC8474513

[ref4] Brakel K. Hook M. A. (2019). SCI and depression: does inflammation commandeer the brain? Exp. Neurol. 320:112977. doi: 10.1016/j.expneurol.2019.112977, PMID: 31203113

[ref5] Cui L. Li S. Wang S. Wu X. Liu Y. Yu W.. (2024). Major depressive disorder: hypothesis, mechanism, prevention and treatment. Signal Transduct. Target. Ther. 9:30. doi: 10.1038/s41392-024-01738-y, PMID: 38331979 PMC10853571

[ref6] Ding Y. Chen Q. (2023). The NF-kappaB pathway: a focus on inflammatory responses in spinal cord injury. Mol. Neurobiol. 60, 5292–5308. doi: 10.1007/s12035-023-03411-x 37286724

[ref7] Dolma S. Kumar H. (2021). Neutrophil, extracellular matrix components, and their interlinked action in promoting secondary pathogenesis after spinal cord injury. Mol. Neurobiol. 58, 4652–4665. doi: 10.1007/s12035-021-02443-5, PMID: 34159551

[ref8] Gu Z. Eils R. Schlesner M. (2016). Complex heatmaps reveal patterns and correlations in multidimensional genomic data. Bioinformatics 32, 2847–2849. doi: 10.1093/bioinformatics/btw313, PMID: 27207943

[ref9] Hu X. Xu W. Ren Y. Wang Z. He X. Huang R.. (2023). Spinal cord injury: molecular mechanisms and therapeutic interventions. Signal Transduct. Target. Ther. 8:245. doi: 10.1038/s41392-023-01477-6, PMID: 37357239 PMC10291001

[ref10] Israel A. (2010). The IKK complex, a central regulator of NF-kappaB activation. Cold Spring Harb. Perspect. Biol. 2:a000158. doi: 10.1101/cshperspect.a000158, PMID: 20300203 PMC2829958

[ref11] Jensen P. Ahlehoff O. Egeberg A. Gislason G. Hansen P. R. Skov L. (2016). Psoriasis and new-onset depression: a Danish Nationwide cohort study. Acta Derm. Venereol. 96, 39–42. doi: 10.2340/00015555-2183, PMID: 26086213

[ref12] Khaksari M. Soltani Z. Shahrokhi N. (2017). Effects of female sex steroids administration on pathophysiologic mechanisms in traumatic brain injury. Transl. Stroke Res. 9, 393–416. doi: 10.1007/s12975-017-0588-5, PMID: 29151229

[ref13] Li Y. Ritzel R. M. Khan N. Cao T. He J. Lei Z.. (2020). Delayed microglial depletion after spinal cord injury reduces chronic inflammation and neurodegeneration in the brain and improves neurological recovery in male mice. Theranostics 10, 11376–11403. doi: 10.7150/thno.49199, PMID: 33052221 PMC7545988

[ref14] Lindqvist D. Janelidze S. Hagell P. Erhardt S. Samuelsson M. Minthon L.. (2009). Interleukin-6 is elevated in the cerebrospinal fluid of suicide attempters and related to symptom severity. Biol. Psychiatry 66, 287–292. doi: 10.1016/j.biopsych.2009.01.030, PMID: 19268915

[ref15] Liu Q. Zhang J. Xiao C. Su D. Li L. Yang C.. (2022). Akebia saponin D protects hippocampal neurogenesis from microglia-mediated inflammation and ameliorates depressive-like behaviors and cognitive impairment in mice through the PI3K-Akt pathway. Front. Pharmacol. 13:7419. doi: 10.3389/fphar.2022.927419, PMID: 36110522 PMC9468712

[ref16] Maldonado-Bouchard S. Peters K. Woller S. A. Madahian B. Faghihi U. Patel S.. (2016). Inflammation is increased with anxiety- and depression-like signs in a rat model of spinal cord injury. Brain Behav. Immun. 51, 176–195. doi: 10.1016/j.bbi.2015.08.009, PMID: 26296565 PMC4679693

[ref17] Marwaha S. Palmer E. Suppes T. Cons E. Young A. H. Upthegrove R. (2023). Novel and emerging treatments for major depression. Lancet 401, 141–153. doi: 10.1016/S0140-6736(22)02080-3, PMID: 36535295

[ref18] Müller-Jensen L. Ploner C. J. Kroneberg D. Schmidt W. U. (2021). Clinical presentation and causes of non-traumatic spinal cord injury: an observational study in emergency patients. Front. Neurol. 12:701927. doi: 10.3389/fneur.2021.701927, PMID: 34434162 PMC8380771

[ref20] Ni S. Luo Z. Jiang L. Guo Z. Li P. Xu X.. (2019). UTX/KDM6A deletion promotes recovery of spinal cord injury by epigenetically regulating vascular regeneration. Mol. Ther. 27, 2134–2146. doi: 10.1016/j.ymthe.2019.08.009, PMID: 31495776 PMC6904668

[ref21] Santo C. C.d. E. Fiorin F. D. S. Ilha J. Duarte M. M. M. F. Duarte T. Santos A. R. S. (2019). Spinal cord injury by clip-compression induces anxiety and depression-like behaviours in female rats: the role of the inflammatory response. Brain Behav. Immun. 78, 91–104. doi: 10.1016/j.bbi.2019.01.012, PMID: 30659938

[ref22] Sun S. C. Ganchi P. A. Ballard D. W. Greene W. C. (1993). NF-κB controls expression of inhibitor IκBα: evidence for an inducible autoregulatory pathway. Science 259, 1912–1915. doi: 10.1126/science.8096091, PMID: 8096091

[ref23] Sun X. Jones Z. B. Chen X. M. Zhou L. So K. F. Ren Y. (2016). Multiple organ dysfunction and systemic inflammation after spinal cord injury: a complex relationship. J. Neuroinflammation 13:260. doi: 10.1186/s12974-016-0736-y, PMID: 27716334 PMC5053065

[ref24] Taniguchi K. Karin M. (2018). NF-kappaB, inflammation, immunity and cancer: coming of age. Nat. Rev. Immunol. 18, 309–324. doi: 10.1038/nri.2017.142 29379212

[ref25] Wang H. Zhang R. Qiao Y. Xue F. Nie H. Zhang Z.. (2014). Gastrodin ameliorates depression-like behaviors and up-regulates proliferation of hippocampal-derived neural stem cells in rats: involvement of its anti-inflammatory action. Behav. Brain Res. 266, 153–160. doi: 10.1016/j.bbr.2014.02.046, PMID: 24613238

[ref26] Wu T. Hu E. Xu S. Chen M. Guo P. Dai Z.. (2021). clusterProfiler 4.0: a universal enrichment tool for interpreting omics data. Innovation (Camb) 2:100141. doi: 10.1016/j.xinn.2021.100141, PMID: 34557778 PMC8454663

[ref27] Wu J. Stoica B. A. Luo T. Sabirzhanov B. Zhao Z. Guanciale K.. (2014a). Isolated spinal cord contusion in rats induces chronic brain neuroinflammation, neurodegeneration, and cognitive impairment. Involvement of cell cycle activation. Cell Cycle 13, 2446–2458. doi: 10.4161/cc.29420, PMID: 25483194 PMC4128888

[ref28] Wu A. Zhang J. (2023). Neuroinflammation, memory, and depression: new approaches to hippocampal neurogenesis. J. Neuroinflammation 20:283. doi: 10.1186/s12974-023-02964-x, PMID: 38012702 PMC10683283

[ref29] Wu J. Zhao Z. Sabirzhanov B. Stoica B. A. Kumar A. Luo T.. (2014b). Spinal cord injury causes brain inflammation associated with cognitive and affective changes: role of cell cycle pathways. J. Neurosci. 34, 10989–11006. doi: 10.1523/jneurosci.5110-13.2014, PMID: 25122899 PMC4131014

[ref30] Yang W. Li J. Zhang M. Yu H. Zhuang Y. Zhao L.. (2022). Elevated expression of the rhythm gene NFIL3 promotes the progression of TNBC by activating NF-κB signaling through suppression of NFKBIA transcription. J. Exp. Clin. Cancer Res. 41:67. doi: 10.1186/s13046-022-02260-1, PMID: 35180863 PMC8855542

[ref31] Ye C. Huang X. Tong Y. Chen Y. Zhao X. Xie W.. (2025). Overexpression of ALKBH5 alleviates LPS induced neuroinflammation via increasing NFKBIA. Int. Immunopharmacol. 144:113598. doi: 10.1016/j.intimp.2024.113598, PMID: 39571266

[ref32] Yu H. Lin L. Zhang Z. Zhang H. Hu H. (2020). Targeting NF-kappaB pathway for the therapy of diseases: mechanism and clinical study. Signal Transduct. Target. Ther. 5:209. doi: 10.1038/s41392-020-00312-6 32958760 PMC7506548

[ref33] Zeng H. Cheng L. Lu D. Z. Fan S. Wang K. X. Xu L. L.. (2023). Unbiased multitissue transcriptomic analysis reveals complex neuroendocrine regulatory networks mediated by spinal cord injury-induced immunodeficiency. J. Neuroinflammation 20:219. doi: 10.1186/s12974-023-02906-7, PMID: 37775760 PMC10543323

[ref34] Zhang Y. Gao Y. Jiang Y. Ding Y. Chen H. Xiang Y.. (2023). Histone demethylase KDM5B licenses macrophage-mediated inflammatory responses by repressing Nfkbia transcription. Cell Death Differ. 30, 1279–1292. doi: 10.1038/s41418-023-01136-x, PMID: 36914768 PMC10154333

[ref35] Zhang C. Zhao S. Huang Z. Xue A. Liu H. Dai S.. (2025). Macropinocytosis enhances foamy macrophage formation and cholesterol crystallization to activate NLRP3 inflammasome after spinal cord injury. Redox Biol. 79:103469. doi: 10.1016/j.redox.2024.103469, PMID: 39700693 PMC11723182

