# Supplementary material for: Nfkbia-driven neuroinflammatory pathways mediate depression following spinal cord injury
Source: Front Mol Neurosci. 2025 Sep 4;18:1596649. doi: 10.3389/fnmol.2025.1596649 (PMC12443535; doi:10.3389/fnmol.2025.1596649)
Supplement: Supplementary file 1 [file Table_1.DOCX]

Supplementary Material


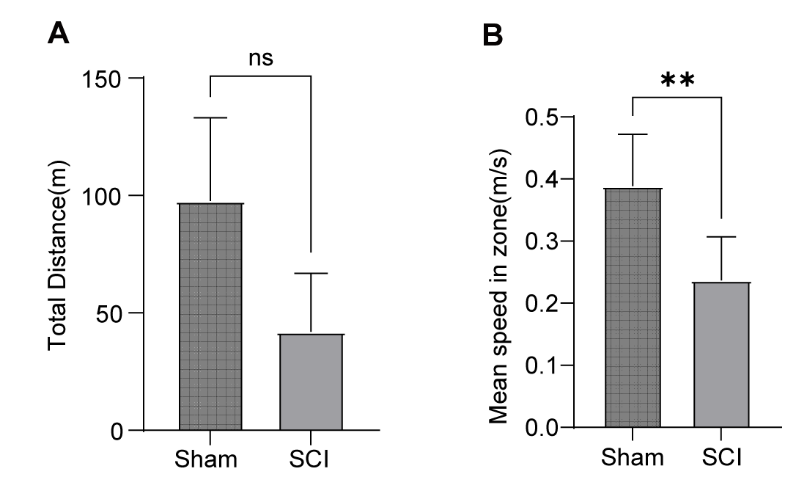


**Supplementary Figure1.** **(A)** Total distance traveled in the OP by Sham and SCI groups. **(B)** Velocity in the OP by Sham and SCI groups. Data are mean ± SD (n = 3); m: meters; ns: not significant; ^**^*P* < 0.01.
